# *Celsr2* Knockout Alleviates Inhibitory Synaptic Stripping and Benefits Motoneuron Survival and Axon Regeneration After Branchial Plexus Avulsion

**DOI:** 10.1007/s12035-022-03198-3

**Published:** 2023-01-03

**Authors:** Lingtai Yu, Mengfan Liu, Fuxiang Li, Qianghua Wang, Meizhi Wang, Kwok-Fai So, Yibo Qu, Libing Zhou

**Affiliations:** 1 Guangdong-Hongkong-Macau CNS Regeneration Institute of Jinan University, Key Laboratory of CNS Regeneration (Jinan University)-Ministry of Education, Guangzhou, 510632 People’s Republic of China; 2 Department of Neurology and Stroke Center, The First Affiliated Hospital & Clinical Neuroscience Institute of Jinan University, Guangzhou, 510632 People’s Republic of China; 3 Neuroscience and Neurorehabilitation Institute, University of Health and Rehabilitation Sciences, Qingdao, 266071 Shandong People’s Republic of China; 4 Co-Innovation Center of Neuroregeneration, Nantong University, Jiangsu, People’s Republic of China

**Keywords:** Brachial Plexus Avulsion, Celsr2, MHC I, Axon Regeneration, Synaptic Stripping, Spinal Motoneurons

## Abstract

Axotomy-induced synaptic stripping modulates survival and axon regeneration of injured motoneurons. Celsr2 is supposed to mediate homophilic interactions of neighboring cells during development, and its role in synaptic stripping remains unknow. In a model of brachial plexus avulsion, *Celsr2* knockout improved functional recovery, motoneuron survival, and axon regeneration. Celsr2 was indicated to express in spinal motoneurons, excitatory and inhibitory interneurons, astrocytes, and a subset of oligodendrocytes using *Celsr2*^*LacZ*^ mice. Double immunostaining showed that the coverage of inhibitory and excitatory vesicles on injured motoneurons were remarkably reduced after injury, whereas more inhibitory vesicles were maintained in *Celsr2*^−/−^ mutants than control mice. In the ultrastructure, the density of inhibitory F-boutons on injured motoneurons was higher in *Celsr2*^−/−^ mutants than controls. Conditional knockout of *Celsr2* in astrocytes or oligodendrocytes showed the similar axotomy-induced synaptic withdrawal to the control. RNAseq of injured spinal samples identified 12 MHC I molecules with significant changes between *Celsr2*^−/−^ and control mice. After injury, expression of MHC I surrounding injured motoneurons was increased, particularly high in *Celsr2*^−/−^ mutants. In conclusion, *Celsr2* knockout enhances MHC I signaling, alleviates inhibitory synaptic stripping cell-autonomously, and contributes to motoneuron survival and regeneration, and Celsr2 is a potential target for neural repair.

**Supplementary Information:**

The online version contains supplementary material available at 10.1007/s12035-022-03198-3.

## Introduction

Spinal motoneurons serve as the final arbiters of skeletal muscle contraction and relaxation via neuromuscular junctions (NMJs) and receive extensive direct and indirect inputs from premotor neurons, and their activities are modulated by excitatory and inhibitory synapses [1, 2]. Modulators of motoneuron activity include inputs from the glutamatergic descending projections, proprioceptive fibers of dorsal root ganglions, GABAergic and glycinergic spinal interneurons [3]. The balance of the excitatory and inhibitory neuronal circuits in the spinal cord is required for maintaining the motor control and influences neural repair after injury or degeneration.

Peripheral axotomy of motoneurons induces synaptic plasticity, characterized by the transient detachment (synaptic stripping) of excitatory and inhibitory boutons on membranes and proximal dendrites of injured motoneurons and the long-term reorganization of neural network in spinal cord [4]. The synaptic stripping is widely reported after the axotomy of hypoglossal nerve [5, 6], abducens nerve [7, 8], gastrocnemius nerve [9], and facial nerve [10, 11]. The plastic changes of synapses contribute to downregulating excitotoxicity in injured motoneurons and initiating neuronal regeneration [12]. Glial cells are supposed to influence synaptic stripping. After axotomy, astrocytes and microglia are recruited and activated, and their protrusions remove presynaptic terminals on injured motoneurons [13]. The interaction between activated astrocytes and axotomized motoneurons regulated by the Stat3 signaling influences coverage of excitatory synapses [14], and the interaction of activated microglia and axotomized motoneurons contributes to eliminating the stripped membranous-vesicular fragments [15].

Immune molecules of MHC I complex are important modulators in synaptic stripping [16]. The absence of MHC I molecule expression preferentially promotes withdrawal of inhibitory presynaptic terminals on injured spinal motoneurons, and this elevates the remaining excitatory/inhibitory ratio and is detrimental to regeneration [17]. IFN1β administration upregulates the expression MHC I molecules in spinal cord to result in better axonal outgrowth and functional recovery after sciatic nerve injury [18]. These studies indicate that the modulation of synaptic stripping is a potential strategy for neural repair.

Brachial plexus avulsion (BPA) usually interrupts the connections of spinal roots with the spinal cord, in which spinal motoneurons show the proximal axon injury. In contrary to the axotomy of distal nerves, BPA triggers a more severity of axon dying back and, eventually, extensive death of motoneurons in the ventral horn [19]. Promoting the survival and axon regeneration of injured motoneurons is a challenge for neural repair after BPA. Like the distal axotomy, root avulsion also induces synaptic stripping of injured spinal motoneurons. In rats with experimental autoimmune encephalomyelitis, the limited inflammation influenced synaptic plasticity as well as survival of lesioned spinal motoneuron after lumbar root avulsion [20]. After L5–L6 ventral root avulsion, a prolonged treatment of brain-derived neurotrophic factor (BDNF) preferentially restores inhibitory synaptic covering on injured motoneurons, conferred injured motoneurons toward a dominance of inhibition, which promotes neuronal survival by downregulating glutamatergic excitotoxicity [21]. Similarly, G-CSF (granulocyte colony stimulating factor) treatment prevents the withdrawal of inhibitory synaptic terminals after ventral root avulsion in rats [22]. These studies propose that the excitatory state of injured motoneurons after root avulsion by modulating synaptic coverage is closely associated with neural survival and repair.

*Celsr2* is one mammalian orthologue of the *drosophila* planar cell polarity (PCP) gene, *flamingo*, which encodes an atypical cadherin receptor containing a seven-transmembrane domain, extracellular cadherin, and EGF repeats and a long intracellular motif [23]. In flies, Flamingo is expressed in adjacent membranes of neighboring cells to regulate intercellular communication via homophilic-interaction. Mutation of *flamingo* gene may interrupt the inter-neuronal interactions and results in the particular phenotype, such as dendrite overlapping in homologous multiple dendritic neurons [24]. Genetic animal studies show that Celsr2 is important for brain development [25], and the phenotypes of impaired neuronal migration and cilia organization in *Celsr2* mutant mice are somehow reminiscent of the deficits in intercellular interaction [26, 27].

After root avulsion, synaptic plasticity induced by axotomy basically indicates the process of interaction changes between injured motoneurons and their afferent neurons. Celsr2 is proposed to be an important modulator for inter-neuronal connections. Therefore, we wonder whether Celsr2 is involved in the synaptic stripping, injured motoneuron survival, and axon regeneration after BPA. To address these issues, we made BPA models to compare synaptic stripping, neuronal survival, and transcriptional profiling in the ventral horns, and studied motor axon regeneration and functional recovery in the mouse model after BPA combined with motor root reimplantation, in control and *Celsr2*^−/−^ mice. In addition, we generated mice with conditional inactivation of *Celsr2* in astrocytes or oligodendrocytes and investigated synaptic stripping after BPA.

## Materials and Methods

### Animals

Animal experiments were carried out according to the Guide for the Care and Use of Laboratory Animals of the National Institutes of Health and approved by the Laboratory Animal Ethics Committee at Jinan University. The generation of *Celsr2*^−/−^ and *Celsr2*^*LacZ*^ mice was described previously [27]. *Celsr2*^−/−^ and *Celsr2* “floxed” mice (*Celsr2*^*f/f*^) were crossed with *Aldh1l1-CreER*^*T2*^ [28] or *Plp-CreER*^*T2*^ [29] mice to generate *Aldh1l1–CreER*^*T2*^;*Celsr2*^*f/*−^ or *Plp-CreER*^*T2*^*;Celsr2*^*f*−^ animals, which were induced by tamoxifen (1 mg/day; Cat No.: T5648, Sigma) for 10 consecutive days (3 days before and 7 days after injury) to inactive *Celsr2* in astrocytes or oligodendrocytes respectively. Animals were housed at 23 ± 1 °C, 12-h dark/light cycle.

### BPA Models

BPA models were made with young adult mice (2–3 months old, 24–28 g) as described [30]. Briefly, C5–C7 spinal segments were exposed under an operating microscope following unilateral hemilaminectomy; right C5–C7 dorsal and ventral roots were dissected out; 2–3 mm of spinal roots in segments of C5 and C7 were removed to prevent reconnection to spinal cord, and the right C6 ventral root was re-implanted to its initial location. During the operation, a thermostatic surgical pad was used to maintain normal body temperature. After the procedure, animals were housed in individual cages. They resumed drinking and eating within 24 h and recovered uneventfully. Animals with either sex were used.

### Behavioral Tests

Behavioral tests were carried out by an experimenter blind to mouse genotypes. All animals were tested 3 days before and 3, 7, 14, 21, 30, 45 days after BPA.

#### Grooming Test

The scores of grooming tests were assessed as reported before [31]. In each test, the forelimb movements were recorded for 5 min with a video camera and two tests were done in the morning and the afternoon. The highest scores were considered as the value of one trial.

#### Climbing Tests

Individual mice were placed in a clear Perspex cylinder (170 mm in height, 90 mm in diameter) and the times of forelimb touching the wall were counted during a 5-min video recording. The ratio of hits by forelimbs on the injured versus intact side was calculated. Each animal was tested for two times, in the morning and the afternoon, and the average of touching times was represented for one trial.

#### Catwalk

Mice walked in an enclosed walkway and images of footprints were recorded. The criteria for data collection were: completing one walk in 0.5–10 s and walking speed variation less than 60%. Main parameters of walking were collected automatically using the software (Noldus, The Netherlands), including max contact area, foot patterns, mean intensity, stride length, stride width, print area, swing speed and swing. Each animal was tested for 3 times and the average of each parameter was calculated. The footprints of forelimbs were plotted.

### Histology and Immunohistochemistry

C5–C7 spinal segments or musculocutaneous nerves were cut into 15-µm-thick frozen sections for immunostaining. The blocking buffer was composed of 5% goat serum and 3% bovine serum albumin diluted in 0.1 M phosphate buffer saline (PBS). Signal was detected with Alexa fluor 546 or 488 coupled secondary antibodies (1:1000, Invitrogen). Primary antibodies were: goat anti- choline acetyltransferase (ChAT, 1:500, ab144p, Millipore), chicken anti-*β*-gal (1:500, ab9361, Abcam), rabbit anti-Calretinin (1:300, ab702, Abcam), mouse anti-Parvalbumin (1:1000, Mab1572, Millipore), rabbit anti-CAMKII (1:500, ab104224, Abcam), rabbit anti-vesicular GABA transporter (VGAT; 1:800, NO131013, Synaptic Systems), mouse anti-vesicular glutamate transporter 1 (vGlut1; 1:1000, Mab5502, Millipore), rat anti-major histocompatibility complex 1 (MHC1; 1:300, sc-59199, Santa Cruz), rabbit anti-glial fibrillary acidic protein (GFAP; 1:1000, AB7260, Abcam), rabbit anti-Iba1(1:1000, 019–19,741, Wako), and rabbit anti-Oligo2 (1:500, ab9610, Merck Millipore).

On day 50 after BPA, the biceps were collected and 7-µm horizontal sections were prepared with a sliding microtome (Leica, Germany) and double stained with rabbit anti-NF200 (1:500, n4142, Sigma) and α-BT (1:1000, Molecular probes, USA) to visualize neuromuscular junctions (NMJs).

### Cell Counting and Cell Density Measurement

Each spinal cord block included 6 serials of alternative transverse sections, and immunostaining for ChAT, GFAP, and Iba1 was performed using one serial of sections respectively. Images were captured under a × 20 objective microscope. ChAT-positive motoneurons were counted in the ventral horn, and GFAP- and Iba1-positive cells were counted in a 0.5 × 0.5 mm^2^ area of the ventral horn respectively. The average from all sections (about 10–15) represented one sample.

### Linear Density of Bouton Coverage

Analyzing synaptic bouton coverage on spinal motoneurons referred to the previous report [32]. Briefly, in immunostained sections, images were captured under a × 63 oil objective using confocal microscope (Zeiss 700, Germany). The ImageJ (NIH) was used to trace the perimeter of each motoneuron and to measure vGlut1- and VGAT-immunoreactive signal, and a plot of perimeter luminance versus location on the perimeter was made accordingly. The luminance peaks 10% above the average luminance of the perimeter were identified as vGlut1- and VGAT-positive terminals. Feret’s diameters of all terminals were calculated, and the ratio of the diameter sum to the motoneuron perimeter was presented as the linear density of bouton coverage.

### Electron Microscopy

Musculocutaneous nerves 50 days after BPA and spinal samples 7 days after BPA were prepared for electron microscopy *(*EM) studies. Briefly, animals were perfused with 2.5% glutaraldehyde (sigma) plus 2% paraformaldehyde, 2-mm distal musculocutaneous nerves or ventral horns of C5–C7 spinal segments were collected for post-fixation at 4 °C overnight. Under dissection microscope, spinal tissues including ventral horn were trimmed into columns and transverse panels were identified for cutting. After washing in PBS, samples were immersed in 0.5% osmic acid, dehydrated in ethanol, and embedded in resin (EMbed 812, Electron Microscope Sciences). Semi-thin (500 nm) transverse sections were stained with 1% Toluidine Blue and images were captured under a 63 × oil objective. For ultrastructural analysis, 50-nm ultrathin sections were prepared for lead staining and the images were captured using a Philips 400 transmission electron microscope. In musculocutaneous nerves, the number of different-sized axons and G-ratio (the inner/the outer diameter of the myelin sheath) were measured using ImageJ.

In spinal sections, motoneurons with large cell bodies (> 35 μm in diameter) were photographed at × 9700 using a transmission electron microscope, and presynaptic terminals on motoneuron membranes were classified as F-type (flattened vesicles) and S-type (spherical vesicles), representing inhibitory and excitatory synapses respectively [17]. The boutons in 100-μm motoneuron membrane were counted. Three animals were used in each group, and 2 well-identified neurons were analyzed in each sample.

### Western Blots

Protein extracts from C5–C7 ventral horns of adult animals 7 days after surgery were analyzed on 10% sodium dodecylsulfate polyacrylamide gels and then transferred to 0.45 μm nitrocellulose membranes. The following primary antibodies were used: rabbit anti-cleavage Capase3 (1:1000; Cat No. 9661, Cell Signaling Technology), rat anti-MHC1 (1:500; sc-59199, Santa Cruz), rabbit anti-GAPDH (1:1,0000; Cat No. 5174, Cell Signaling Technology), anti-β-tubulin (1:10,000; rabbit, Cat No. 2146, Cell Signaling Technology); the secondary antibodies included Peroxidase anti-rabbit IgG (1:5,000, ab6721, Abcam) and peroxidase anti-mouse IgG (1:10,000; Vector Laboratories). Immunoreactivity was detected using an enhanced chemiluminescence (ECL) detection kit (1,705,061, Bio-Rad).

### Quantitative Real-Time PCR

Three days after tamoxifen induction, cervical spinal segments were collected, and total RNA was extracted using the TRIzol kit (Invitrogen). cDNA was synthesized from total 1 μg RNA using the Reverse Transcription System (Promega) and 1 μl cDNA subjected to PCR using the Eco™ Real-Time PCR System (illumina). The cycling condition was 35 cycles of 95 °C for 5 min, 95 °C for 30 s, 60 °C for 30 s, and 72 °C for 40 s. The expression levels of *Celsr2* were evaluated using the 2-ΔΔCt method, and GAPDH was used as a reference. All reactions were performed in triplicate and repeated 3 times. The primers included: 5′

-CACGATGGCCTGAGGGTTT-3′ (Celsr2 forward), 5′-CCTTGTGGAGAAAGGTGTCCT-3′ (Celsr2 reverse), 5′-CCAATGTGTCCGTCGTGGATCT -3′ (GAPDH forward), 5′-GTTGAAGTCGCAGGAGACAACC-3′ (GAPDH reverse).

### RNAseq

Three days after BPA, ventral horns of C5–C7 spinal segments were separated for RNA extraction using the TRIzol Plus RNA Purification Kit (Cat. No. 12183018A, Invitrogen Life Technologies). About 8 μg RNA from each sample was submitted for transcriptome sequencing using HiSeq 2000 (Illumina Hiseq 2000, BGI) platforms. Gene expression was calculated according to fragments per kilobase of exon per million fragments mapped (FPKM), and differentially expressed genes (DEGs) were identified using Poisson distribution analysis with FDR ≤ 0.001 and the absolute value of log_2_Ratio ≥ 1. A hypergeometric test was used for gene ontology (GO) enrichment analysis for mapping all DEGs to terms in the GO database [33]. The pathway analysis was based on the KEGG database as report [34].

### Statistical Analysis

Data were presented as mean ± SEM. Results were analyzed with two-way ANOVA or Student *t* test of two-independent samples. The cell count and fluorescence density were assessed using ImageJ software. The significant level was set as *P* < 0.05.

## Results

### Celsr2 Constitutive Knockout Improves Functional Recovery in Mice with BPA

**Fig. 1:**
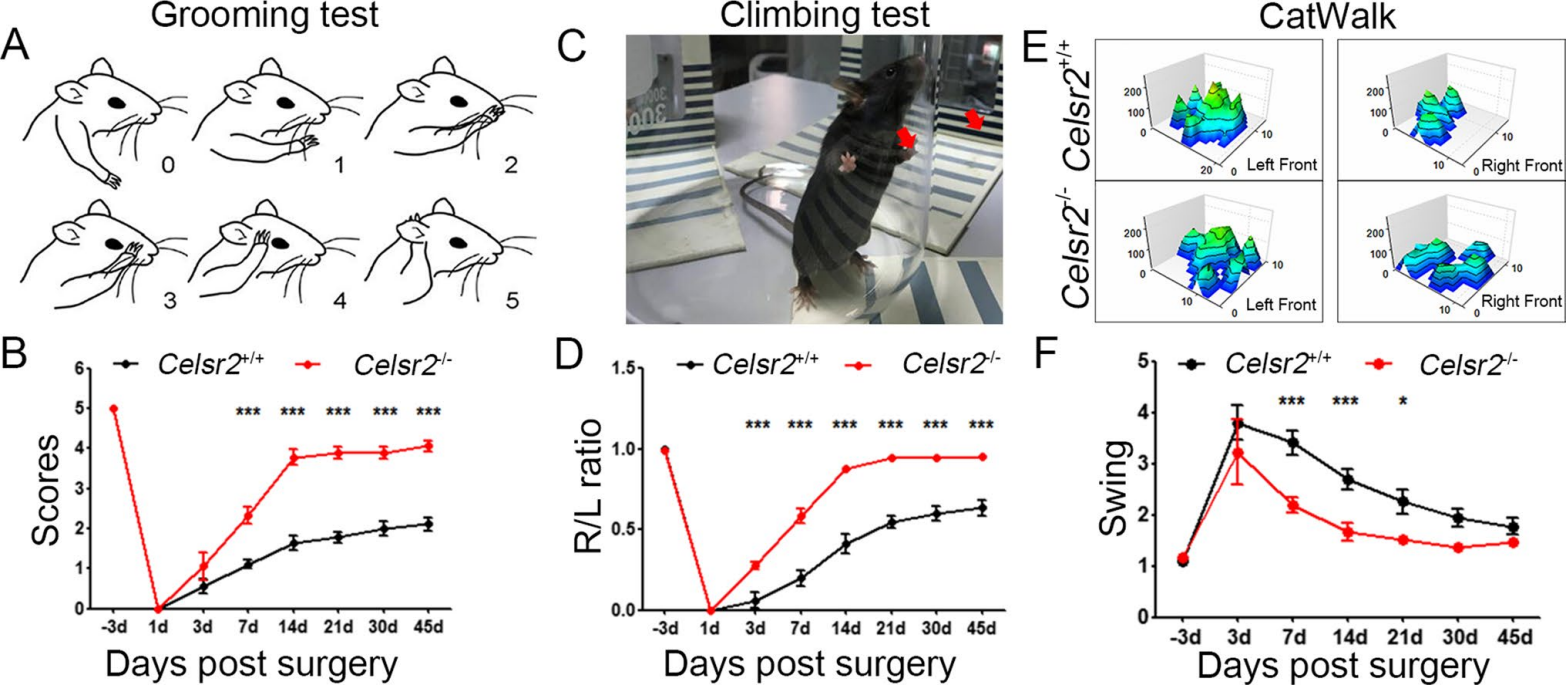
*Celsr2* constitutive knockout improves functional recovery after BPA with motor root reimplantation. **A**, **B** Scores of the grooming test (**A**) show the significant increase at day 7, 14, 21, 30, and 45 after surgery in *Celsr2*^−/−^ mice compared to control mice (**B**). **C**, **D** The usage of forelimbs is assessed by climbing tests (**C**), showing the ratio of injured side to intact side (R/L ratio) is significantly increased in mutants compared to controls (**D**). **E**, **F** After surgery, Catwalk tests show that the forepaw prints on the injured side (right panel in **E**) are separated well in *Celsr2*.^−/−^ mutants but blurred in control mice (**E**), and the ratio of swing time (injured side to intact side) is decreased (closer to 1) at day 7, 14 and 21 in the mutant compared to the control (**F**). *P < 0.05; ***P < 0.001; two-way ANOVA with Sidak’s multiple comparisons, *n* = 9

To study the effect of *Celsr2* constitutive knockout on neural repair and functional recovery after injuries, we established C5–C7 spinal root avulsion followed with C6 motor root re-implantation using young adult animals [30]. In this model, regenerating spinal axons can re-innervate biceps brachii following the re-implanted motor roots and restore function [35]. We assessed functional recovery of the elbow flexion using the grooming test. Before surgery, *Celsr2*^−/−^ and control mice displayed comparable scores with a mean of 5 (Fig. 1A). The animals with the scores more than 0 at day 1 post surgery were excluded. The animals exhibited gradual functional recovery from day 7 post surgery (Fig. 1B). The mean score displayed a sharp increase from 3 to 14 days and a slower increase thereafter (Fig. 1B). At day 45 post surgery, the mean score was 4.05 ± 0.13 in the *Celsr2*^−/−^ and 2.11 ± 0.16 in the control (*P* < 0.001, *n* = 9 in each group), indicating the significantly better recovery in the mutant.

The spontaneous movement of the elbow joint was assessed by the climbing test (Fig. 1C). During the 5-min test, we counted the number of forepaws touching the glass wall and calculated the ratio of right (injured side) to left (intact side) forepaws. After surgery, the ratios were gradually increased, faster in the mutant than the control, which reached 0.95 ± 0.02 in the *Celsr2*^−/−^ versus 0.64 ± 0.05 in the control (*P* < 0.001, *n* = 9 in each group) at day 45 post surgery (Fig. 1D).

In Catwalk tests, the prints of the palm and fingers of left intact forepaws were easily identified in both groups as indicated in tri-dimension (3D) footprint intensity charts (Fig. 1E), whereas the prints of mutant injured right forepaws were better separated than those in the control (Fig. 1E) at day 45 post surgery. Coordinating walk was evaluated by the swing and the swing speed, representing the time ratio and the speed of the forelimb lifting away and touching the floor respectively. The swing speed was comparable in two groups, but the swing (time ratio) was significantly lower at day 7, 14, 21, and 30 in the *Celsr2*^−/−^ compared to the control (Fig. 1F; *P* < 0.001 at day 7 and 14, *P* < 0.05 at day 21, *n* = 9 in each group).

### Celsr2^−/−^ Mice Have Better Motoneuron Survival and Axon Regeneration After BPA

**Fig. 2:**
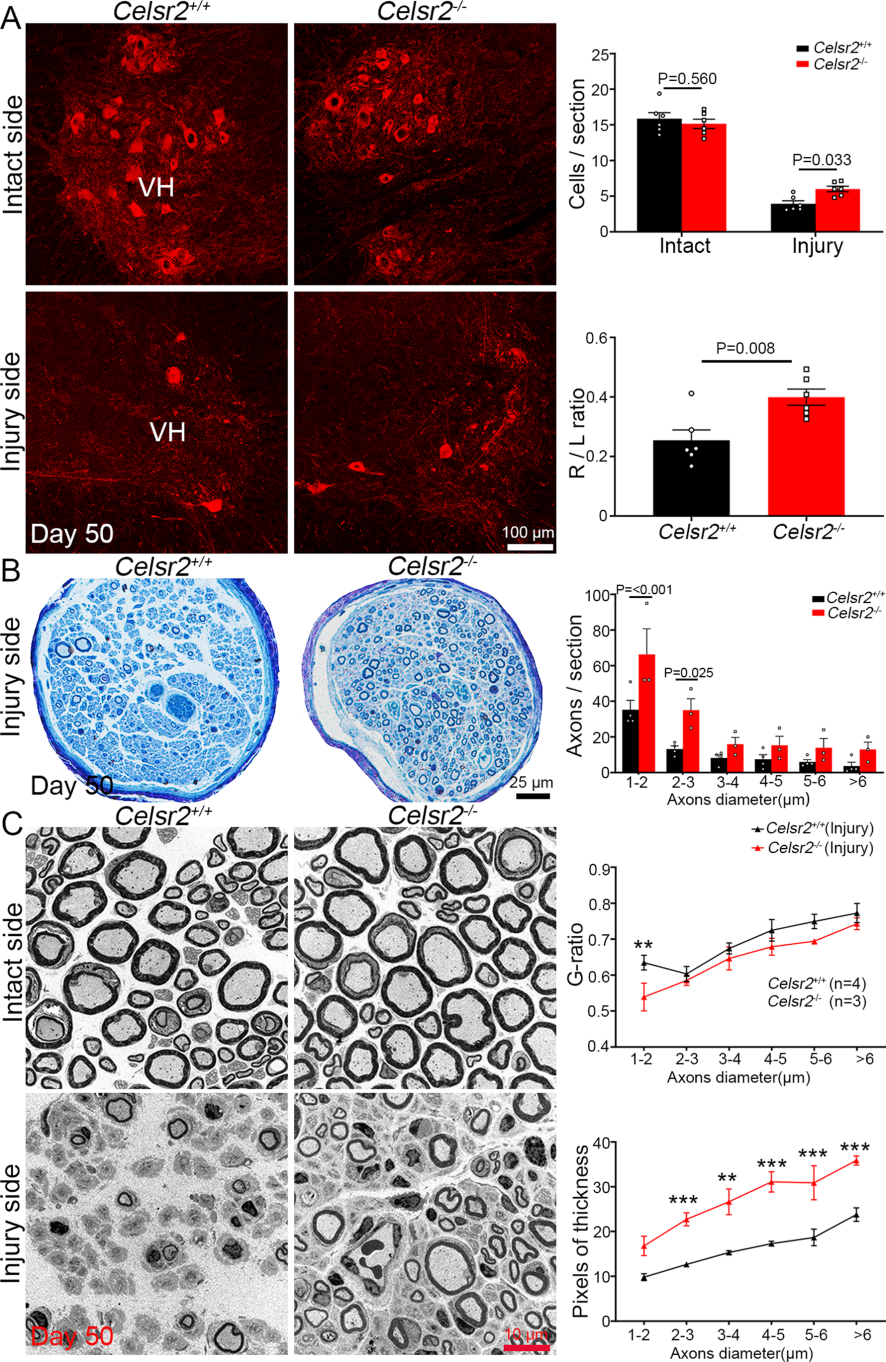
Celsr2 negatively influences neuron survival and axon regeneration. **A** Anti-ChAT immunostaining with C5–C7 spinal sections day 50 after surgery shows the numbers of spinal motoneurons in the ventral horn (VH) are comparable in two groups on intact sides (*P* > 0.05), higher in the mutant than the control on injured sides (*P* < 0.05). The survival of injured motoneurons is indicated by the ratio of ChAT-positive neurons on injury sides (R) to intact sides (L), showing a significant increase (*P* < 0.01). Unpaired two-tailed Student’s *t* test, *n* = 6 animals in each group. **B** In semi-thick sections of musculocutaneous nerves, Toludine blue staining show the increase of axon numbers, particularly in small axons (1–3 µm in diameter) in the mutant compared to the control on injury sides (*P* < 0.001 or 0.05). Unpaired two-tailed Student’s *t* test, *n* = 4 in the control and 3 in the mutant. **C** Under EM, the G-ratio of myelinated axons in musculocutaneous nerves is similar in two groups on intact sides (L), but decreased in 1–2 µm-diameter small axons in the mutant on injured sides (R). The pixels of myelin sheath thickness are increased in the mutant compared to the control on injured sides. **P < 0.01; ***P < 0.001; two-way ANOVA with Sidak’s multiple comparisons, *n* = 4 in the control and 3 in the mutant

To provide morphological evidence of functional recovery after BPA, we collected C5–C7 spinal segments and performed anti-ChAT immunostaining on frozen sections. In the ventral horn, at day 50, ChAT-immunoreactive motoneurons were visible on the intact side, whereas a few ChAT-positive neurons were preserved on the injury side (Fig. 2A). Statistical analysis showed the number was comparable in two groups on the intact side (control and *Celsr2*^−/−^ in cells/section: 15.9 ± 0.8 and 15.2 ± 0.7, *P* > 0.05, *n* = 6 in each group), higher in the *Celsr2*^−/−^ compared to the control on the injured side (control and mutant in cells/section: 3.9 ± 0.4 and 6.0 ± 0.4, *P* < 0.05, *n* = 6 in each group), and the motoneuron survival ratio was significantly increased in the *Celsr2*^−/−^ (control and mutant: 0.25 ± 0.04 and 0.40 ± 0.03, *P* < 0.01, *n* = 6 in each group). To study the process of axotomy-induced spinal motoneuron death, we collected injured spinal segments at day 7, 14, and 21 after BPA and performed anti-ChAT immunostaining to count survived motoneurons on injury sides (Supplementary Fig. 1A). In the control, the numbers showed a gradual decrease from day 7 to 21 with a significant difference between day 14 and day 7, but not between day 21 and 14 (Supplementary Fig. 1B, C). In the *Celsr2*^−/−^, the decrease was significant from day 14 to day 21, but not remarkable between day 7 and 14 (Supplementary Fig. 1B). More spinal motoneurons were preserved at day 14 and 21 in the *Celsr2*^−/−^ compared to the control on injury sides respectively (Supplementary Fig. 1B), but the numbers of spinal motoneurons were comparable on intact sides at each timepoint in two groups (data not shown). The comparisons of survival ratios at each timepoint indicated the slower neuronal death in the *Celsr2*^−/−^ (Supplementary Fig. 1C; control and *Celsr2*^−/−^: 0.44 ± 0.05 and 0.55 ± 0.01 at day 7, 0.34 ± 0.04 and 0.53 ± 0.02 at day 14, 0.24 ± 0.04 and 0.39 ± 0.04 at day 21). Western blots showed the protein levels of cleaved Caspase3 were lower in mutant injured spinal samples at day 7 (Supplementary Fig. 1D, E). The findings indicate *Celsr2* knockout slows down BPA-induced neuronal death.

To assess the regeneration ability of surviving motoneurons, we collected musculocutaneous nerves at day 50 post surgery and prepared semi-thin sections for toluidine blue staining. Axon numbers were comparable in two groups on intact sides (data not shown). On injured sides, there were more axons in the *Celsr2*^−/−^ compared to the control, particularly in small-diameter axons (Fig. 2B; control and mutant in axons/section: 35.25 ± 5.30 and 66.33 ± 14.33 in 1–2 µm-diameter axons, 13.25 ± 1.75 and 35.00 ± 6.43 in 2–3 µm diameter axons, *P* < 0.001 and *P* < 0.05 respectively, *n* = 4 and 3 in the control and the mutant). In EM sections (Fig. 2C), myelin sheath of regenerated axons was assessed by analyzing the G-ratio (inner/outer diameter of axons) of musculocutaneous nerves, showing comparable between two groups on intact sides (data not shown), and smaller in 1–2 µm small axons in the *Celsr2*^−/−^ compared to the control on injured sides (Fig. 2C; control and mutant: 0.63 ± 0.02 and 0.54 ± 0.04, *P* < 0.05, *n* = 4 in each group). In addition, the thickness of myelin sheath was significantly increased in the mutant in different-size axons on injured sides (Fig. 2C; *P* < 0.001 or 0.01, *n* = 4 in each group).

At day 50 after BPA, biceps showed atrophy in two groups on injured sides, and their wet weight was comparable on intact sides in two groups but higher in the *Celsr2*^−/−^ than the control on injured sides (Supplementary Fig. 2A–D). In biceps horizontal sections, anti-NF200 and a-BT double staining showed that many newly-formed NMJs were visible in the mutant, but rare in the control, at day 50 after BPA (Supplementary Fig. 2E, F).

### Celsr2 Constitutive Knockout Slows Down Inhibitory Synaptic Stripping on Injured Spinal Motoneurons

Axotomy-induced synaptic stripping modulates the excitatory state of injured motoneurons and imposes an effect on neuronal survival and axon regeneration [12]. During neural development, *Celsr* orthologue *flamingo* was reported to regulate cell-to-cell interaction [24]. In the ventral horn, motoneurons receive inhibitory and excitatory inputs from spinal segmental interneurons. Our previous studies showed that all spinal motoneurons were expressed by Celsr2 [36]. Using *Celsr2*^*LacZ*^ mice, in which Celsr2 expression could be visualized by β-gal signal, we found that Parvalbumin-positive, Calretinin-positive GABAergic, and CamKII-positive glutamatergic interneurons co-expressed Celsr2 in the ventral horn using double immunostaining (Supplementary Fig. 3). Therefore, we asked whether *Celsr2* was involved in synapse withdrawal after BPA and analyzed the coverage of presynaptic terminals on spinal motoneuron membranes.

**Fig. 3:**
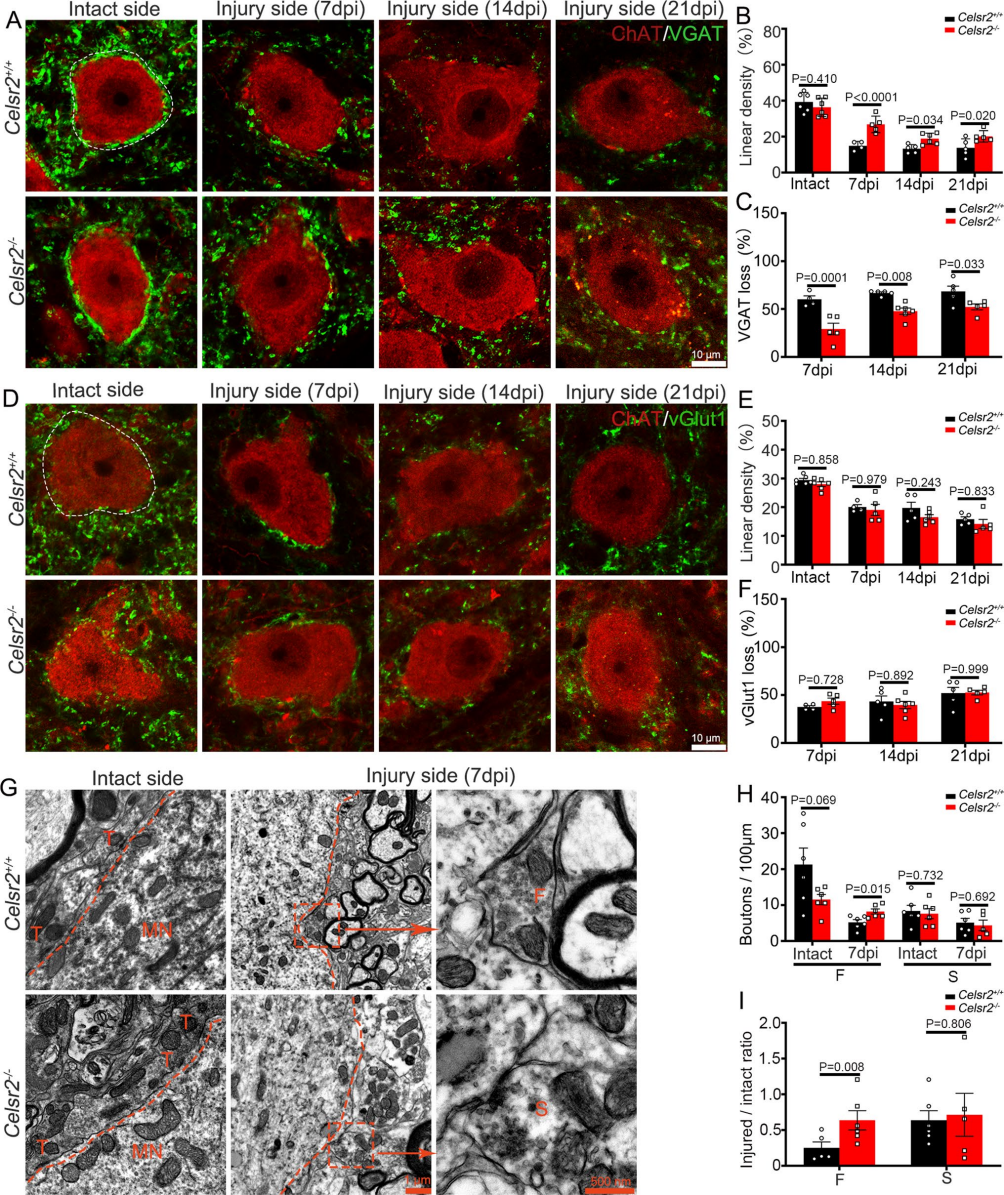
*Celsr2* inactivation slows down inhibitory synaptic stripping on injured motoneurons. C5–C7 spinal sections are for double immunostaining at day 7, 14, and 21 after BPA, and for EM studies on day 7 after BPA. **A**–**C** Anti-VGAT (green) and -ChAT (red) double immunostaining shows that the coverage of VGAT-immunoreactivity on motoneuron membranes decreases on injured sides compared to intact sides in two groups (**A**). The linear density of VGAT immunoreactivity is comparable in two groups on intact sides (*P* > 0.05), but significantly increased at day 7, 14, and 21 on injured sides in the mutant compared to the control (**B**; *P* < 0.0001 at day 7, *P* < 0.05 at day 14 and 21). Normalized to intact sides, the VGAT loss is decreased in the mutant compared to the control at three timepoints after BPA (*P* < 0.01 at day 7 and 14, *P* < 0.05 at day 21; **C**). **D**–**F** Anti-vGlut1 (green) and -ChAT (red) double immunostaining shows that the coverage of vGlut1-immunoreactivity on motoneuron membranes decreases on injured sides compared to intact sides at day 7, 14, and 21 in two groups (**D**). Linear density is comparable in two groups on both sides (E, *P* > 0.05). Normalized to intact sides, the vGlut1 loss is similar in two groups (F, *P* > 0.05). **G**–**I** Ultrastructure of ventral horns under EM shows motoneuron (MN) membrane, presynaptic terminals (T), F- and S- boutons 7 days after BPA in two groups (**G**). The number of F-boutons within 100-µm motoneuron membrane is significantly increased in the mutant on injured sides (*P* < 0.01), but comparable in two groups on intact sides (*P* > 0.05); the density of S-Boutons shows no differences in two groups (*P* > 0.05) (**H**). The bouton density ratio (injured to intact sides) shows an increase of F-boutons (*P* < 0.01), but not of S-boutons (*P* > 0.05), in the mutant compared to the control (**I**). Two-way ANOVA with Sidak’s multiple comparisons; immunofluorescent staining: *n* = 4 animals in the control and 5 animals in the mutant at day 7, *n* = 5 animals in the control and 6 animals in the mutant at day 14, *n* = 5 animals/group at day 21; EM study: *n* = 6 neurons from 3 animals in each group; *dpi* days post injury

Transverse spinal sections of C6 segment were processed for anti-VGAT immunostaining to disclose inhibitory synaptic vesicles 7, 14, and 21 days after BPA (Fig. 3A). On intact sides, the coverage of VGAT-positive immunoreactivity on motoneuron membranes was relatively stable at different timepoints after injury. Compared to intact sides, the coverage was significantly decreased at day 7, 14, and 21 on injured sides in two groups (Fig. 3A), indicating a phenotype of inhibitory synapse stripping. Statistical analysis showed that the linear density of VGAT-positive vesicles on motoneurons was comparable in two groups on intact sides (Fig. 3B; control and mutant: 39.2 ± 2.2% and 36.3 ± 2.1%, *P* > 0.05, 6 animals in each group), significantly higher in the *Celsr2*^−/−^ than the control on injured sides at day 7 (control and mutant: 14.9 ± 1.2% and 26.8 ± 2.1%, *P* < 0.0001, 4 animals in the control and 5 animals in the mutant), day 14 (control and mutant: 13.3 ± 1.0% and 18.8 ± 1.3%, *P* < 0.05, 5 animals in the control and 6 animals in the mutant), and day 21 (control and mutant:13.8 ± 0.1% and 20.2 ± 1.4%, *P* < 0.05, 5 animals in each group) after BPA (Fig. 3B). Normalized to intact sides, the percentage of VGAT-positive vesicle loss was less in *Celsr2*^−/−^ mice compared to control mice on injured sides (Fig. 3C; control and mutant: 60.1 ± 3.7% and 29.2 ± 6.1% at day 7, 66.7 ± 1.2% and 47.8 ± 3.3% at day 14, 68.4 ± 5.5% and 52.3 ± 3.1% at day 21, *P* < 0.01 at day 7 and 14, *P* < 0.05 at day 21). Using anti-vGlut1 immunostaining to label excitatory vesicles, remarkable withdrawal of excitatory synaptic terminals on spinal motoneurons happened on day 7, 14, and 21 after BPA compared to the intact sides (Fig. 3D). Unexpectedly, the coverage of vGlut1-positive particles was indiscriminate on intact sides or injured sides in control and *Celsr2*^−/−^ mice by assessing the linear density and vGlut1-positive vesicle loss (Fig. 3E, F; *P* > 0.05). In ultrastructure images, inhibitory and excitatory boutons are readily characterized by F and S types respectively [17], and the densities of F-type, but not S-type, boutons on spinal motoneurons were significantly higher on injured sides 7 days after BPA in the *Celsr2*^−/−^ compared to the control (Fig. 3G, H; control and mutant: 5.2 ± 0.8 and 8.2 ± 0.7 boutons in 100 µm, *P* < 0.01, *n* = 6 neurons from 3 animals in each group), and the ratio (injured/intact) of F-boutons was also significantly increased in the mutant compared to the control (Fig. 3I; control and mutant: 0.3 ± 0.1 and 0.8 ± 0.1, *P* < 0.01, *n* = 6 neurons from 3 animals in each group).

**Fig. 4:**
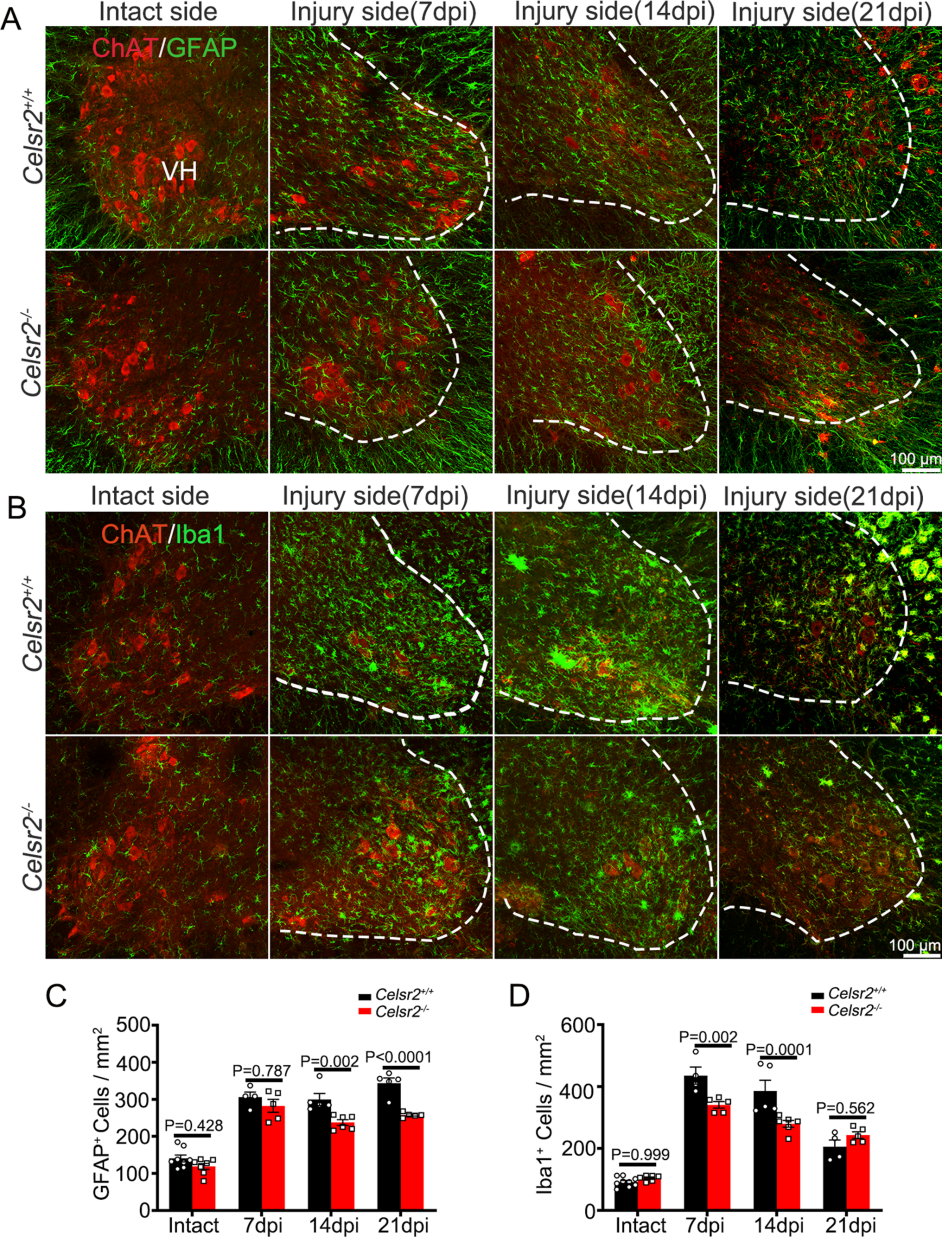
Glial response is alleviated in *Celsr2*.^−/−^ injured spinal cords. **A**, **B** Spinal sections of C5–C7 segments are collected 7, 14, and 21 days post injury (dpi) for double immunostaining of anti-ChAT (red) and -GFAP (green) or anti-ChAT (red) and -Iba1 (green), showing the increase of reactive proliferating astrocytes and microglia (green) surrounding axotomized spinal motoneurons in the ventral horn (VH). **C**, **D** Statistic show the densities of GFAP-positive cells in the ventral horn are decreased in the mutant compared to the control on injury sides at 14 and 21 dpi (C, *P* < 0.01). The densities of Iba1-positive cells are decreased in the mutant on injured sides at 7 and 14 dpi (D, *P* < 0.01). Two-way ANOVA with Sidak’s multiple comparisons, *n* = 4 mice in the control and 5 mice in the mutant at day 7, *n* = 5 mice in the control and 6 in the mutant at day 14, *n* = 5 mice in each group at day 21

These findings indicate that *Celsr2*^−/−^ injured spinal motoneurons maintain more inhibitory synaptic inputs to limit excitatory toxicity, and this process could be indirectly reflected by glial response. At day 7, 14, and 21, C5–C7 spinal sections were immunostained with anti-GFAP and anti-Iba1 antibodies, and abundant proliferating astrocytes and microglia accumulated in the ventral horn on injured sides (Fig. 4A, B). The astrocyte density was significantly lower in the *Celsr2*^−/−^ than the control on injured sides at day 14 and 21 after BPA (Fig. 4C; control and mutant in cells/mm^2^: 300 ± 16 and 237 ± 8, *P* < 0.01, *n* = 5 mice in the control and 6 mice in the mutant at day 14; 344 ± 14 and 258 ± 3, *P* < 0.0001, *n* = 5 mice in each group at day 21), and the density decrease was also present in microglial cells on injured sides at day 7 and 14, but not at day 21 after BPA (Fig. 4D; control and mutant in cells/mm^2^: 435 ± 28 and 341 ± 11 at day 7, 385 ± 35 and 280 ± 11 at day 14, *P* < 0.01, *n* = 4 mice in the control and 5 mice in the mutant at day 7, *n* = 5 mice in the control and 6 mice in the mutant at day 14).

### Celsr2 Inactivation in Astrocytes or Oligodendrocytes Does Not Affect Synaptic Stripping

**Fig. 5:**
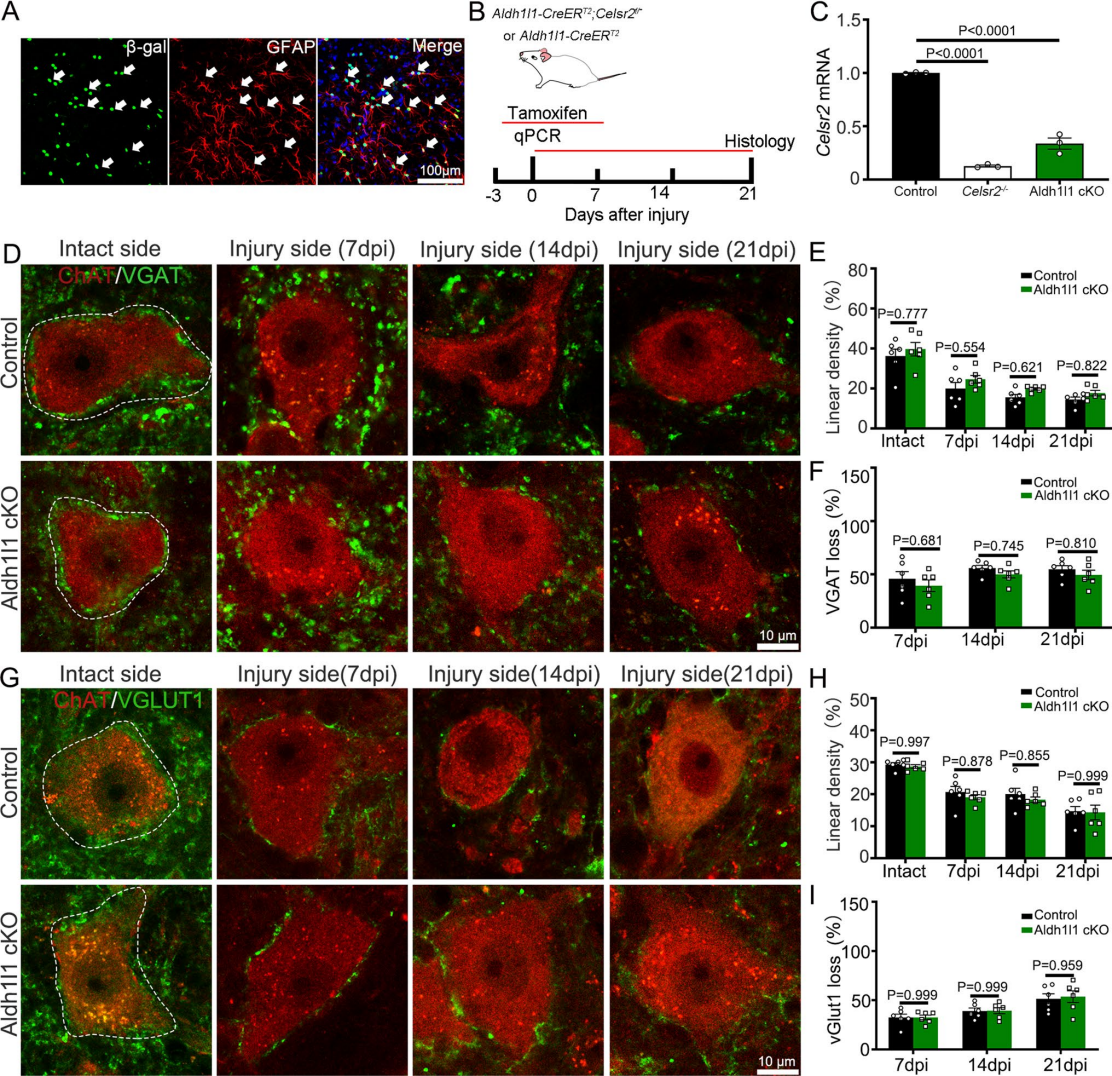
Conditional inactivation of *Celsr2* in astrocytes does not affect synaptic withdrawal after BPA. **A**–**C** In spinal sections of adult *Celsr2*^*LacZ*^ mouse, double immunostaining shows GFAP-positive cells co-express *ß*-gal (arrows, **A**). Conditional inactivation of *Celsr2* in astrocytes and the experimental design are illustrated in the schematic (**B**). Celsr2 mRNA levels in spinal samples are significantly downregulated in *Aldh1l1–CreER*^*T2*^; *Celsr2*^*f* /−^ mice (*Aldh1l1* cKO) upon 3-days tamoxifen induction, but still higher than that in *Celsr2*^−/−^ mice (**C**). **D**–**F** The coverage of inhibitory synaptic vesicles on spinal motoneuron membranes is visualized by anti-VGAT and -ChAT double immunostaining (**D**). The linear densities of VGAT-positive vesicles on both sides and the percentage of VGAT-positive vesicles loss (normalized to intact sides) on injury sides show similar changes in two groups (**E**, **F**; *P* > 0.05). **G**–**I** Anti-vGlut1 and -ChAT double immunostaining shows BPA-induced excitatory synapse withdrawal on spinal motoneuron membranes in two groups (**G**). The reduction is comparable in two groups at day 7, 14, and 21 after BPA (**H**, **I**; *P* > 0.05). Two-way ANOVA with Sidak’s multiple comparisons, *n* = 6 mice at each timepoint in each group; dpi, days post-injury

Axotomy-induced synaptic stripping is the change of inhibitory and excitatory neuron terminals on injured spinal motoneurons. The expression pattern of Celsr2 suggests that it may directly mediate inter-neuronal interactions to influence synaptic stripping as presented above. We then asked whether Celsr2 might indirectly affect synaptic stripping through glial cells. Using *Celsr2*^*LacZ*^ mice, we found that Celsr2 was expressed in GFAP-positive cells in adult spinal cords (Fig. 5A). To conditionally knockout *Celsr2* in astrocytes, we generated *Aldh1l1–CreER*^*T2*^*;Celsr2*^*f/*−^ mice, which were induced by tamoxifen for 10 consecutive days (3 days before and 7 days after BPA) (Fig. 5B). After 3 days of induction, spinal samples were examined by quantitative real-time PCR (RT-qPCR) showing that *Celsr2* mRNA levels were significantly downregulated in *Aldh1l1–CreER*^*T2*^*;Celsr2*^*f/*−^ mice compared to the control, but still higher than that in *Celsr2*^−/−^ mice (Fig. 5C). At day 7, 14, and 21 after BPA, C5–C7 spinal segments were collected. Anti-VGAT and -ChAT double immunostaining showed that the similar decrease of inhibitory synaptic vesicles on spinal motoneuron membranes was observed in two groups at day 7, 14, and 21 respectively (Fig. 5D–F). Excitatory synaptic coverage was assessed by anti-vGlut1 and -ChAT immunostaining. BPA induced the similar decrease of vGlu1-positive boutons on injured motoneurons in two groups at three timepoints respectively (Fig. 5G–I). Thus, *Celsr2* inactivation in astrocytes has no impact on axotomy-induced synaptic stripping.

Similarly, we studied Celsr2 expression in spinal oligodendrocytes and conditionally inactivated *Celsr2* using *Plp–CreER*^*T2*^*;Celsr2*^*f/*−^ mice upon tamoxifen induction (Supplementary Fig. 4A). Celsr2 was expressed in a subgroup of Olig2-positive oligodendrocytes and tamoxifen-induction downregulated *Celsr2* mRNA levels in spinal cords of *Plp–CreER*^*T2*^*;Celsr2*^*f/*−^ mice (Supplementary Fig. 4A–C). Double immunostaining showed the coverage of inhibitory (VGAT-positive) and excitatory (vGlut1-positive) vesicles on injured spinal motoneurons was comparable in two groups at day 7, 14, and 21 respectively (Supplementary Fig. 4D–I), indicating that synaptic withdrawal is not affected by Celsr2 expression in oligodendrocytes.

### Celsr2 Knockout Enhances MHC I Signaling in Injured Spinal Cords

**Fig. 6:**
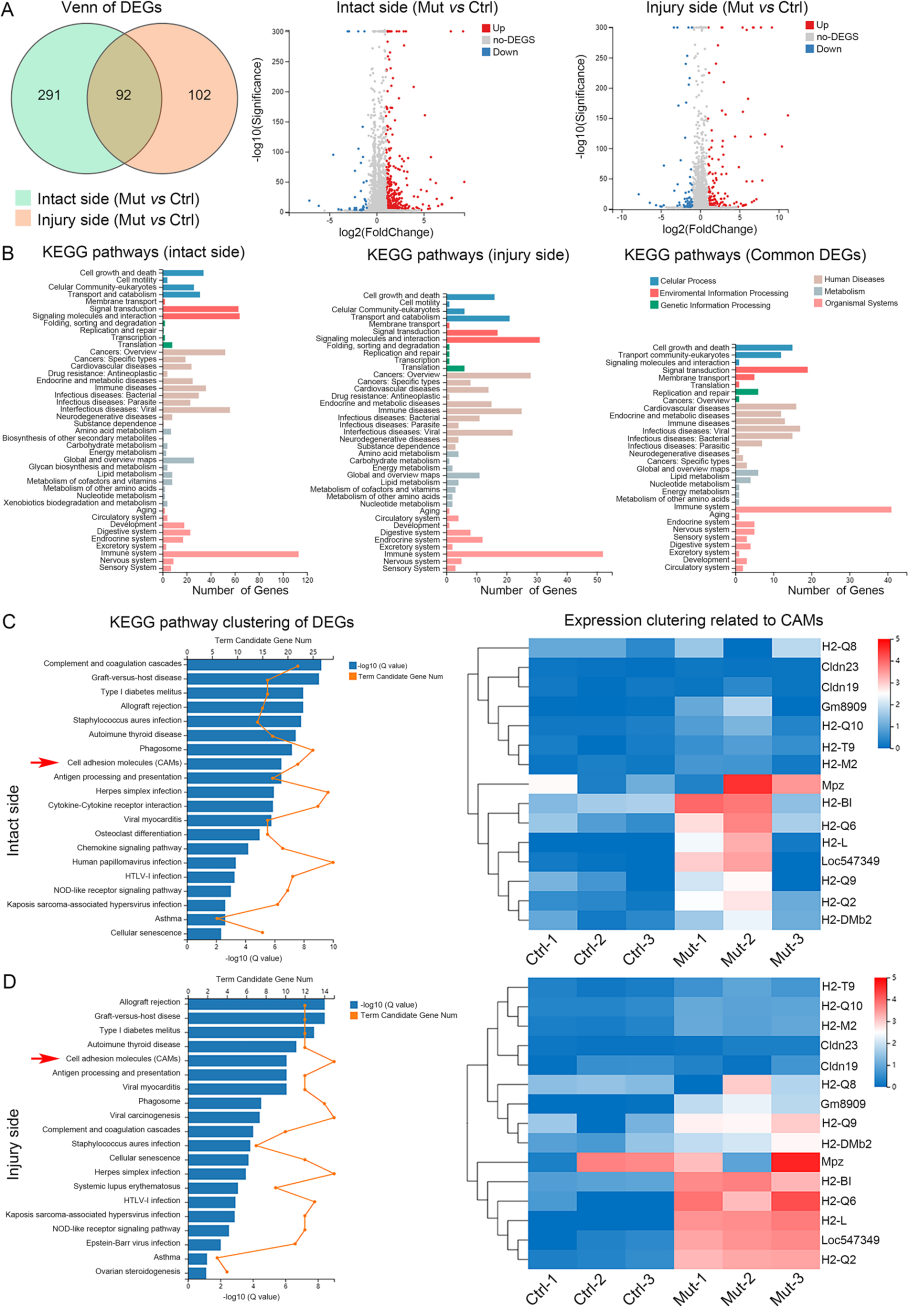
*Celsr2* constitutive knockout results in changes of gene expression in ventral horns after BPA. Three days after BPA, ventral parts of C5–C7 spinal segments were performed for RNAseq. **A** DEGs between the mutant (Mut) and the control (Ctrl) are shown in the Venn graph and the volcano plots. **B** KEEG pathways analysis indicates most abundant DEGs are related the immune system. **C** On intact sides, KEEG pathway clustering of DEGs identifies 15 cell adhesion molecules (CAMs) between mutant and control animals, some of which shows variation in the same group. **D** On injured sides, 12 of 15 CAMs identified by KEEG pathway clustering belong to MHC I molecules, including H2-Q2, Loc547349, H2-L, H2-Q6, H2-BI, H2-DMb2, H2-Q9, Gm8909, H2-Q8, H2-M2, H2-Q10 and H2-T9, and most of them show stable elevation in mutant mice compared to control mice. Ctrl-1, 2, 3 and Mut-1, 2, 3 in **C** and **D** indicate 3 controls and 3 mutants respectively. The blue-to-red color change of light bars in C and D represents the expression levels from low to high

**Fig. 7:**
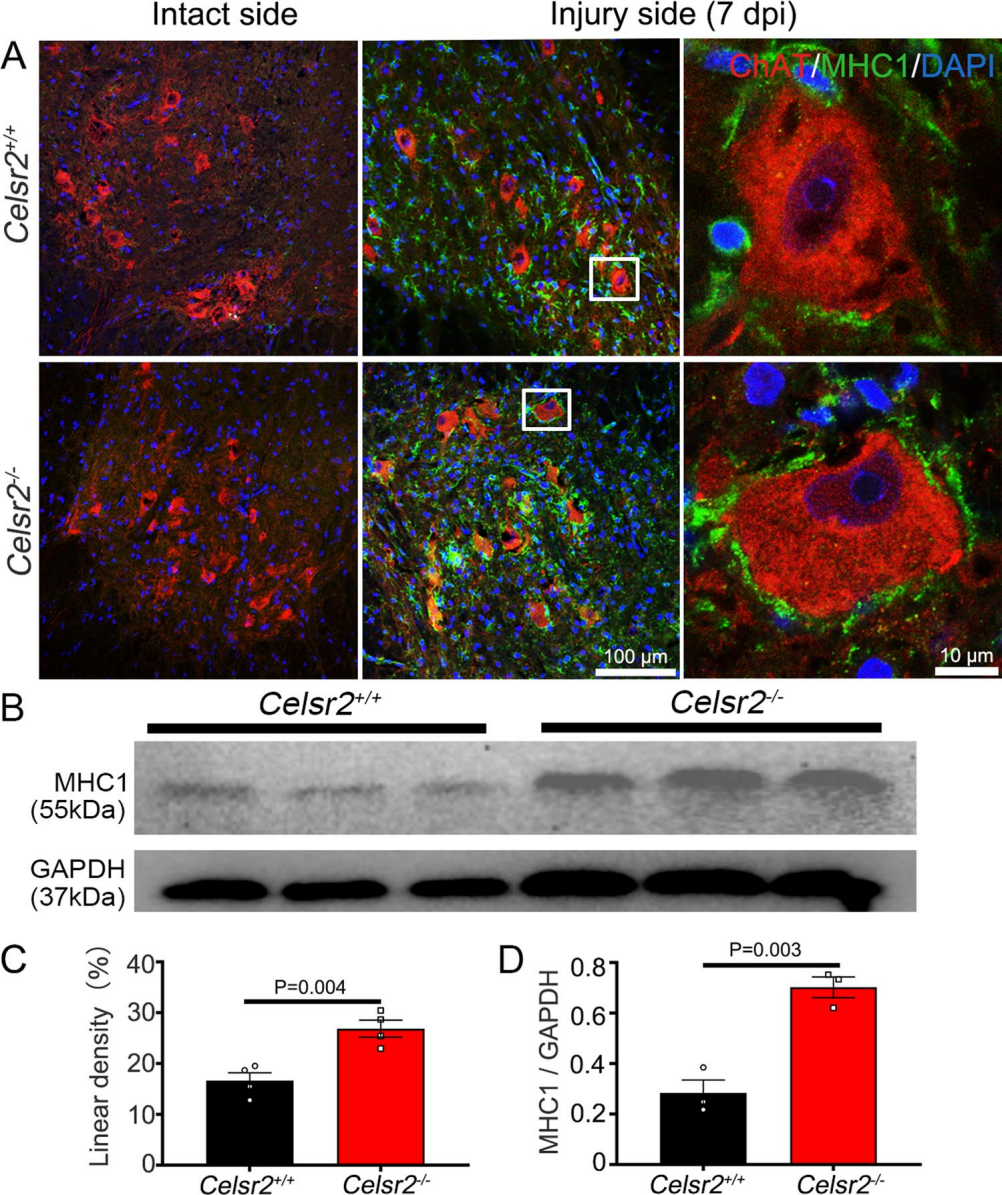
MHC I signaling is elevated in *Celsr2*^−/−^ mutants after BPA. **A** After 7 days post injury (dpi), anti-ChAT (red) and -MHC1 (green) immunostaining are performed with spinal sections of C5–C7 segments. On intact sides, rare MHC1-positive signal is visualized (left panels). On injured sides, axotomized spinal motoneurons are surrounded by the abundant MHC1-immunoreactivity (middle panels), some of which covers the motoneuron membranes (right panels). Selected areas in middle panels are enlarged in right panels respectively. DAPI (blue) counterstains nuclei. **B** In spinal samples 7 dpi, protein levels of MHC1 are assessed by western blots using anti-MHC1 antibodies. Unpaired two-tailed Student’s *t*-test, *n* = 3 animals in each group. **C**, **D** Statistic show the significant increase of the MHC1-immunoractivity coverage (linear density) on axotomized motoneuron membranes (**C**), and of MHC1 protein levels (**D**) in the mutant compared to the control (*P* < 0.01). For linear density comparison, *n* = 48 neurons in the control and 63 neurons in the mutant, 4 animals in each group. Unpaired two-tailed Student’s *t* test

To study the potential mechanism of Celsr2 involved in synaptic stripping, we carried out RNAseq by collecting ventral parts of C5–C7 spinal segments 3 days after BPA. There were 383 differently expressed genes (DEGs) and 194 DEGs identified on intact and injured sides between control and mutant animals respectively, and 92 DEGs in common (Fig. 6A). KEEG pathway analysis showed that the most abundant DEGs were related to the immune system (Fig. 6B). As axotomy-induced synaptic stripping reflects the changes of cell-to-cell interactions, we then focused on cell adhesion molecules of DEGs in KEGG pathway clustering. On intact sides, the relative mRNA levels of 12 MHC I molecules showed the increase trend in the *Celsr2*^−/−^ compared to the control (Fig. 6C), and the changes were more stable in 3 animals of each group on injured sides (Fig. 6D). These DEGs included H2-Q2, Loc547349, H2-L, H2-Q6, H2-BI, H2-Dmb2, H2-Q9, Gm8909, H2-Q8, H2-M2, H2-Q10, and H2-T9. To further confirm this finding, C5–C7 spinal segments were collected 7 days after BPA for anti-MHC I immunostaining. On intact sides, rare MHC I-positive signal was recognized in two groups (Fig. 7A). However, increased MHCI immunoreactivity appeared in the ventral horns and surrounded the preserved motoneuron pools on injured sides (Fig. 7A). Linear density of MHC I immunoreactivity on motoneuron membranes was significantly increased in the *Celsr2*^−/−^ compared to the control (Fig. 7A, C; control and mutant: 16.6 ± 1.5% and 26.9 ± 1.7%, *P* < 0.01, *n* = 47 and 63 neurons in the control and mutant, 4 mice in each group). In Western blots of spinal samples, MHC I protein level was also increased in the *Celsr2*^−/−^ on injured sides 7 days after BPA (Fig. 7B, D; control and mutant in MHCI/GAPDH ratio: 0.28 ± 0.05 and 0.70 ± 0.04, *P* < 0.01, *n* = 3 animals in each group).

## Discussion

Atypical cadherin *Celsr2* is one orthologue of *flamingo*, a member of core PCP genes. Flamingo is supposed to communicate polarity between neighboring cells and direct the formation of polarized structures via homophilic interactions in many contexts [37]. Molecule-mediated cell-to-cell interactions are critical for cellular behaviors involved in many biological processes, such as neuronal migration, self-avoidance of neurite outgrowth, ommatidial formation and epithelial cell organization, and the abnormalities were widely reported in *flamingo* mutation [38–42]. *Celsr2* knockout mice were partially reminiscent of these phenotypes during development, including defective migration of facial motoneurons [26], abnormal cilial organization [27]. In this study, we provide novel evidence that Celsr2 is required for modulating interactions of spinal segmental interneurons and injured motoneurons involved in injured motoneuron survival and axonal regeneration in adults.

BPA is a type of severe axotomy, in which proximal interruption of motor roots with cell bodies results in rapid motoneuron death [43]. Slowing down the progress of motoneuron loss is critical for neural repair. After BPA, we reimplanted C6 motor roots back injured spinal cords to provide a bridge for the axonal regeneration of survived neurons [30]. In this model, *Celsr2*^−/−^ mutants showed better functional recovery of forelimbs in grooming, climbing, and Catwalk tests compared to control mice. The behavioral improvements in mutants were supported by the increase of preserved spinal motoneurons in the ventral horn and regenerating axons in musculocutaneous nerves, more newly-formed NMJs in biceps. These results indicate that *Celsr2* inactivation contributes to the survival and axon regeneration of spinal motoneurons after BPA.

Excitatory toxicity is one of important mechanisms involved in axotomy-induced retrograde motoneuron death and suppressing glutamate toxicity contributes to axotomized motoneuron survival [44]. Spinal motoneurons receive multiple inputs from GABAergic inhibitory and glutamatergic excitatory interneurons, which keep the excitatory/inhibitory balance for maintaining neural activity. Axotomy-induced synaptic stripping of motoneurons is a classic biological phenomenon to modulate the excitatory state of injured neurons by removing presynaptic inputs on injured motoneurons [12]. Celsr2 is an atypical cadherin member and supposed to regulate cellular interactions recognized by long extracellular domains on neighboring cells [45]. In the ventral horn of spinal cords, Celsr2 is expressed in spinal motoneurons, GABAergic inhibitory, and glutamatergic excitatory interneurons, which provides the possibility for Celsr2-mediated interactions of spinal motoneurons and interneurons. In line with the previous reports in axotomies of motoneurons [46], BPA induced the withdrawal of inhibitory and excitatory boutons on injured spinal motoneurons in our study. *Celsr2* constitutive knockout significantly slows down inhibitory, but not excitatory, synaptic stripping on motoneurons after BPA, as indicated in double immunostaining and EM studies. The phenomenon that *Celsr2* inactivation preferentially impacts on inhibitory synaptic withdrawal is similar to the study using *ß2-microglobulin* knockout mice [17], and the modulation of synaptic stripping may be synapse-type specific. In *Celsr2*^−/−^ mutants, injured spinal motoneurons were kept in a more inhibitory state to limit excitatory toxicity, which was also supported by observing less glial response surrounding injured spinal motoneurons and downregulated expression of activated Caspase3 in spinal samples. The decrease of reactive astrocytes in *Celsr2*^−/−^ injured spinal cords was mainly found at day 14 and 21 after BPA, and the direct effect of *Celsr2* inactivation on astrocytes could not be excluded. In our study, *Celsr2*^−/−^ mice have better axon regeneration and functional recovery after BPA, and this outcome may attribute to two explanations: firstly, *Celsr2* inactivation benefits neuronal survival through regulating synaptic stripping in the present work, which alleviates the excitotoxicity on injured motoneurons to facilitate their survival; secondly, *Celsr2* inactivation elevates small GTPases in injured motoneurons to enhance their regeneration ability in the previous report [36, 47].

After BPA, we did not find any differences of the coverage of inhibitory and excitatory boutons on intact spinal motoneurons, indicating that Celsr2 inactivation does not affect synapse formation and maturation on spinal motoneurons. The possible explanation is that the role of Celsr2 on developing interconnections of spinal motoneurons and interneurons is compensated by other Celsr member such as Celsr3 during development as reported [48]. In adults, Celsr2 maintains high expression in spinal cords and plays dominant roles when Celsr3 expression is rare in the central nervous system [49]. In addition, Celsr2 is also expressed in astrocytes and a subset of oligodendrocytes. Conditional knockout of *Celsr2* in astrocytes or oligodendrocytes does not change BPA-induced synaptic stripping. These results indicate that Celsr2 mediates inter-neuronal interactions in a cell-autonomous manner.

The contribution of Celsr2 inactivation to limiting inhibitory synapse stripping on injured spinal motoneurons is also supported by the finding of the elevated expression of MHC I molecules in RNAseq, double immunostaining, and Western blots. MHC I molecules have been shown to be important for activity-dependent synaptic plasticity and remodeling during brain development [50]. Previous studies demonstrated that MHC I molecules were crucial for the selective maintenance of putative inhibitory synapses on axotomized spinal motoneurons after sciatic nerve transection, and lack of MHC I expression impaired axon regeneration of axotomized motoneurons [17]. In our study, we found the most abundant DEGs in KEEG pathway clustering was related to the immune system and identified 12 MHC I molecules with significant elevation in *Celsr2*^−/−^ mutants compared to control animals after BPA. The immunostaining study indicates that motor root avulsion induces MHC I expression surrounding injured spinal motoneurons, and *Celsr2* inactivation significantly enhances injury-induced MHC I expression supported by the coverage of MHC I immunoreactivity on spinal motoneurons and protein levels of spinal samples in Western blots.

Taking together, our study demonstrates that *Celsr2* inactivation selectively limits inhibitory synaptic stripping by elevating MHC I expression, which is agreement with the study in MHC I mutant animals [17], and promotes injured motoneuron survival, axon regeneration, and functional recovery after BPA, the similar improvement of neural repair in mice with IFNβ-induced MHC I expression after sciatic nerve crush [18]. Thus, Celsr2 is a potential modulator for axotomy-induced synaptic stripping and may be a novel target for neural repair after injuries in adults.

## Supplementary Information

Below is the link to the electronic supplementary material.

Supplementary file1 (DOCX 6041 KB)

## Data Availability

The data that support the findings of this study are available from the corresponding author upon reasonable request.
