# Supplementary material for: Celsr2 Knockout Alleviates Inhibitory Synaptic Stripping and Benefits Motoneuron Survival and Axon Regeneration After Branchial Plexus Avulsion
Source: Mol Neurobiol. 2023 Jan 3;60(4):1884–900. doi: 10.1007/s12035-022-03198-3 (PMC9984348; doi:10.1007/s12035-022-03198-3)
Supplement: Supplementary file 1 — Supplementary file1 (DOCX 6041 KB) [file 12035_2022_3198_MOESM1_ESM.docx]

**Supplementary materials**

**Title:** *Celsr2* inactivation alleviates inhibitory synaptic stripping and benefits motoneuron survival and axon regeneration after branchial plexus avulsion

**
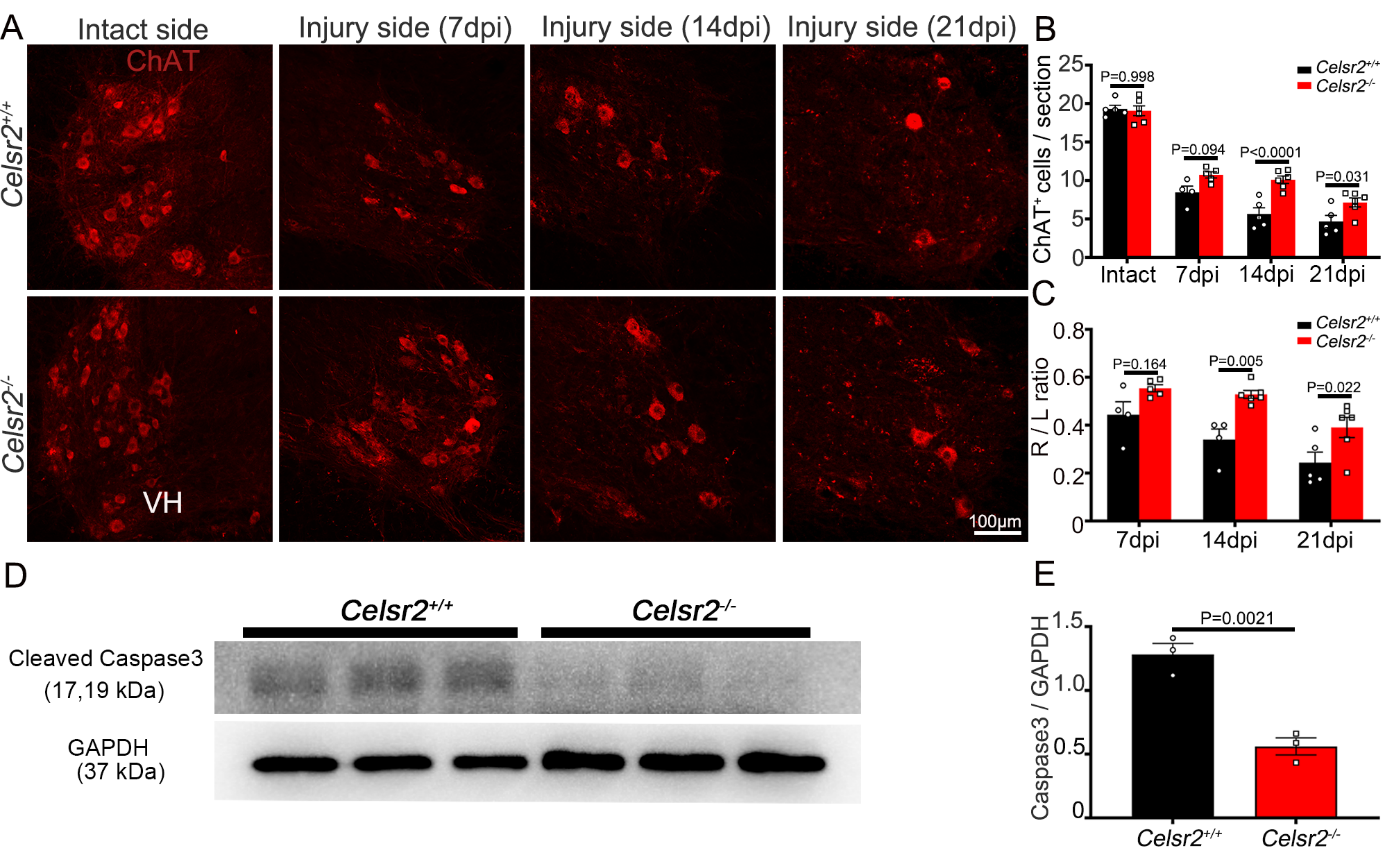
**

**Supplementary Fig. 1 *Celsr2* knockout prevents BPA-induced neuronal loss.**

A-C: Seven-, 14- and 21-days post injury (dpi), spinal sections of C5-C7 segments are immunostained by anti-ChAT antibodies showing the decrease of ChAT-positive neurons on injured sides compared to intact sides in two groups (A). There are more survived motoneurons in the mutant than the control at 7, 14 and 21 dpi on injured sides, but no differences in two groups on intact sides (B). On the injury sides, the numbers of survived motoneurons are comparable at 14 and 21 dpi in the control, at 7 and 14 dpi in the mutant. The neuronal survival is indicated by the neuron ratio of injured sides to intact sides (R/L) showing the significant increase in the mutant compared to the control (C). Unpaired two-tailed Student’s *t*-test, n=5 animals in the control and 6 animals at each timepoint in each group.

D, E: Protein levels of cleaved Caspase3 are assessed by western blots using spinal samples 7 days after BPA (D). Statistic shows a decrease in the mutant compared to the control (E). Unpaired two-tailed Student’s *t*-test, n=3 animals in each group.

**
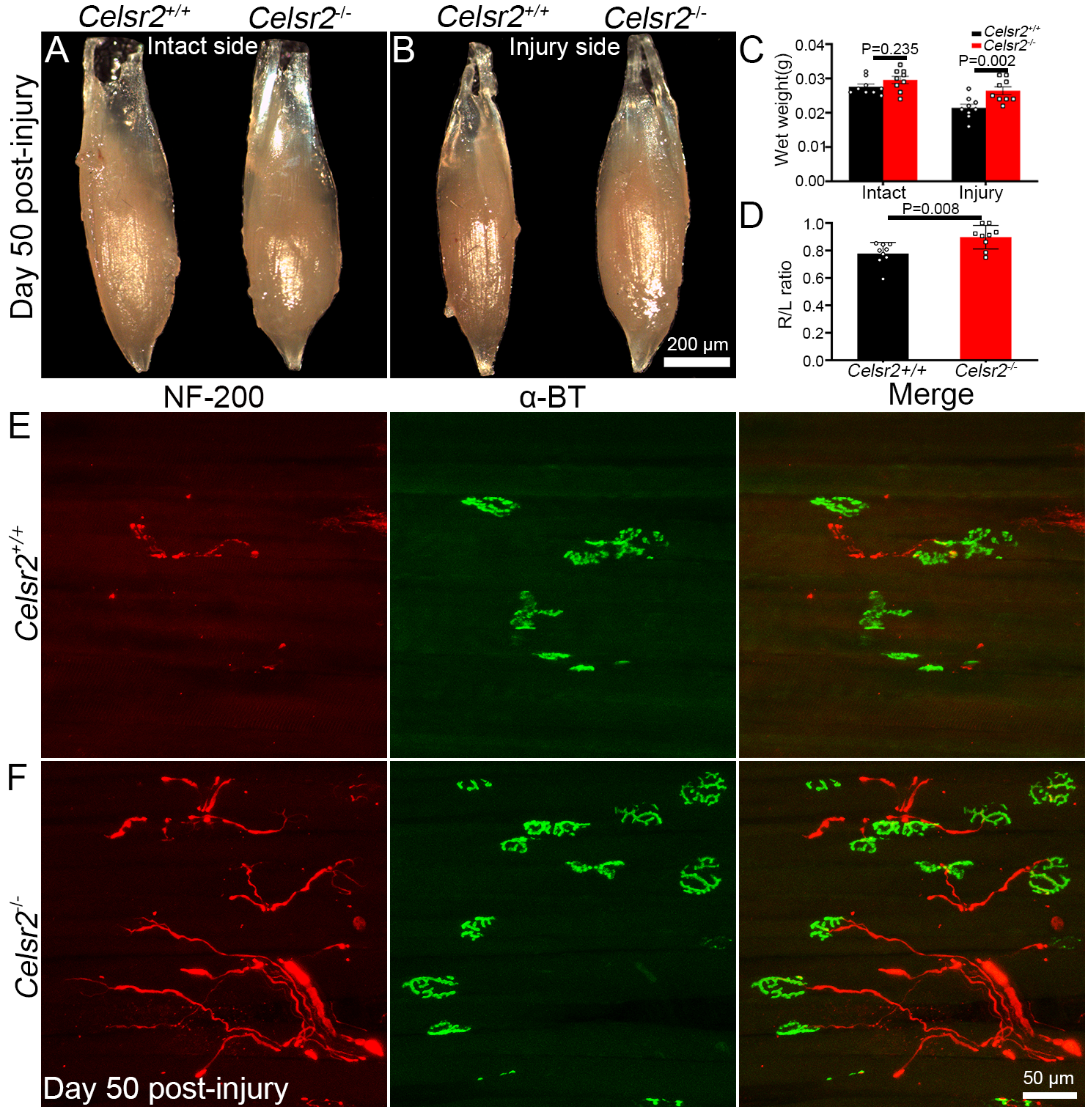
**

**Supplementary Fig. 2 *Celsr2* knockout slows down muscle atrophy and contributes to new NMJs formation after BPA.**

A-D: After 50 days of surgery, biceps show atrophy on injured sides (B) compared to intact sides (A). Wet weight of biceps is increased in the mutant compared to the control on injured sides, whereas there are no differences in two groups on intact sides (C). The weight ratio of injured sides to intact sides (R/L) shows a significant increase in the mutant compared to the control. Unpaired two-tailed Student’s *t*-test, n=9 in each group, n.s., not significant.

E, F: In horizontal sections of biceps, double immunostaining for NF-200 (red) and ɑ-BT (blue) is used to disclose newly-formed NMJs on injured sides day 50 post injury. Some well-formed NMJs are visualized in the mutant (F) whereas rare NMJs could be found in the control (E).


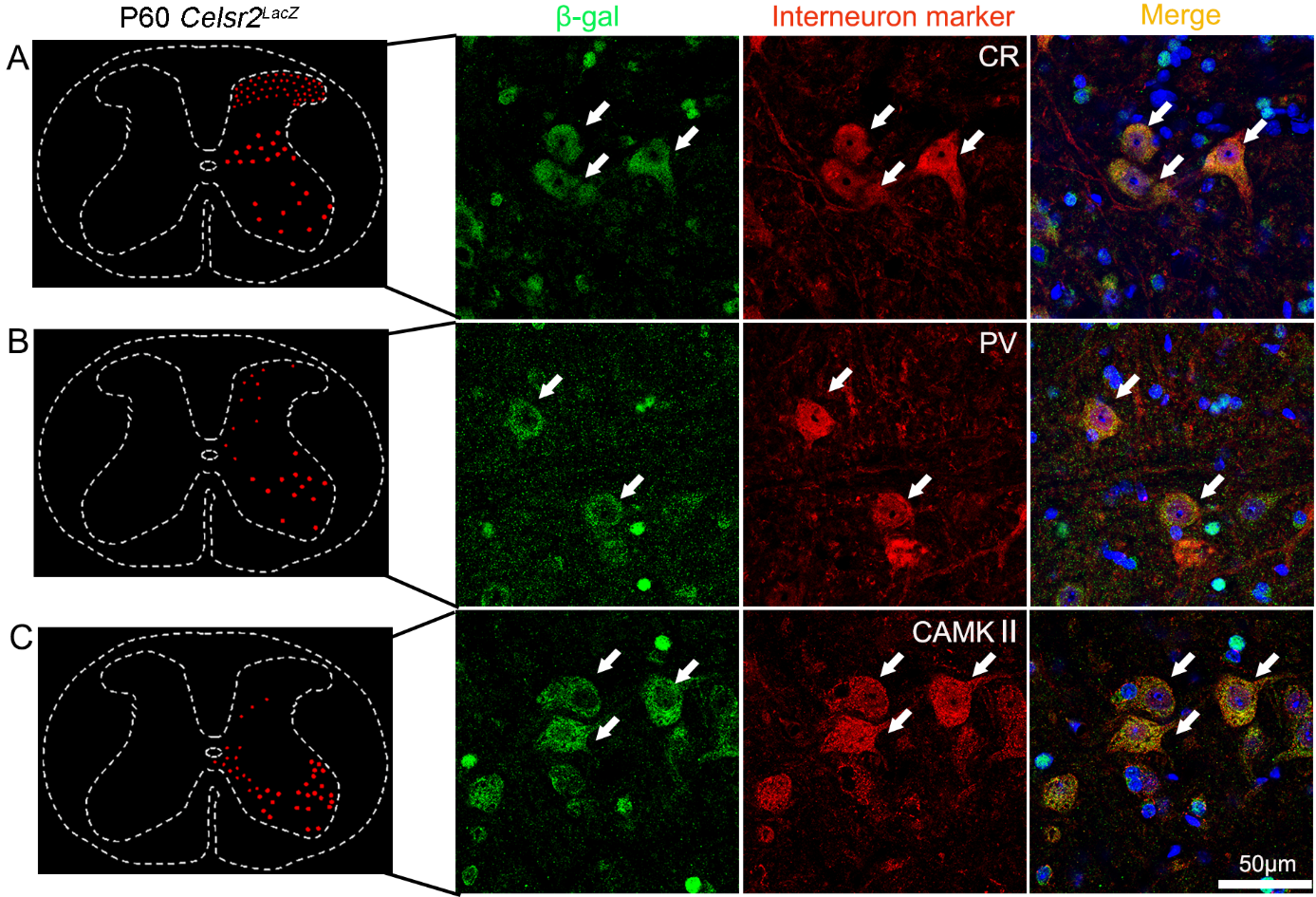


**Supplementary Fig. 3 Celsr2 is expressed in spinal interneurons in adult mice.**

In spinal sections from postnatal day 60 (P60) *Celsr2^LacZ^* mouse, anti-*β*-gal immunostaining (green) indirectly discloses Celsr2 expression.

A: Anti-Calretinin (CR) immunostaining (red) shows the distribution of CR-positive inhibitory interneurons in the gray matter of spinal cord (indicated in schema on the left panel). In the ventral horn, CR-positive interneurons co-express *β*-gal (arrows).

B: Double immunostaining for *β*-gal and Parvalbumin (PV) shows PV-positive inhibitory interneurons co-express *β*-gal in the ventral horn (arrows). Shema on the left panel indicates the distribution of PV-positive interneurons.

C: Double immunostaining for *β*-gal and CAMKII shows glutamatergic excitatory interneurons co-express *β*-gal in the ventral horn (arrows). Shema on the left panel indicates the distribution of CAMKII-positive interneurons.

**
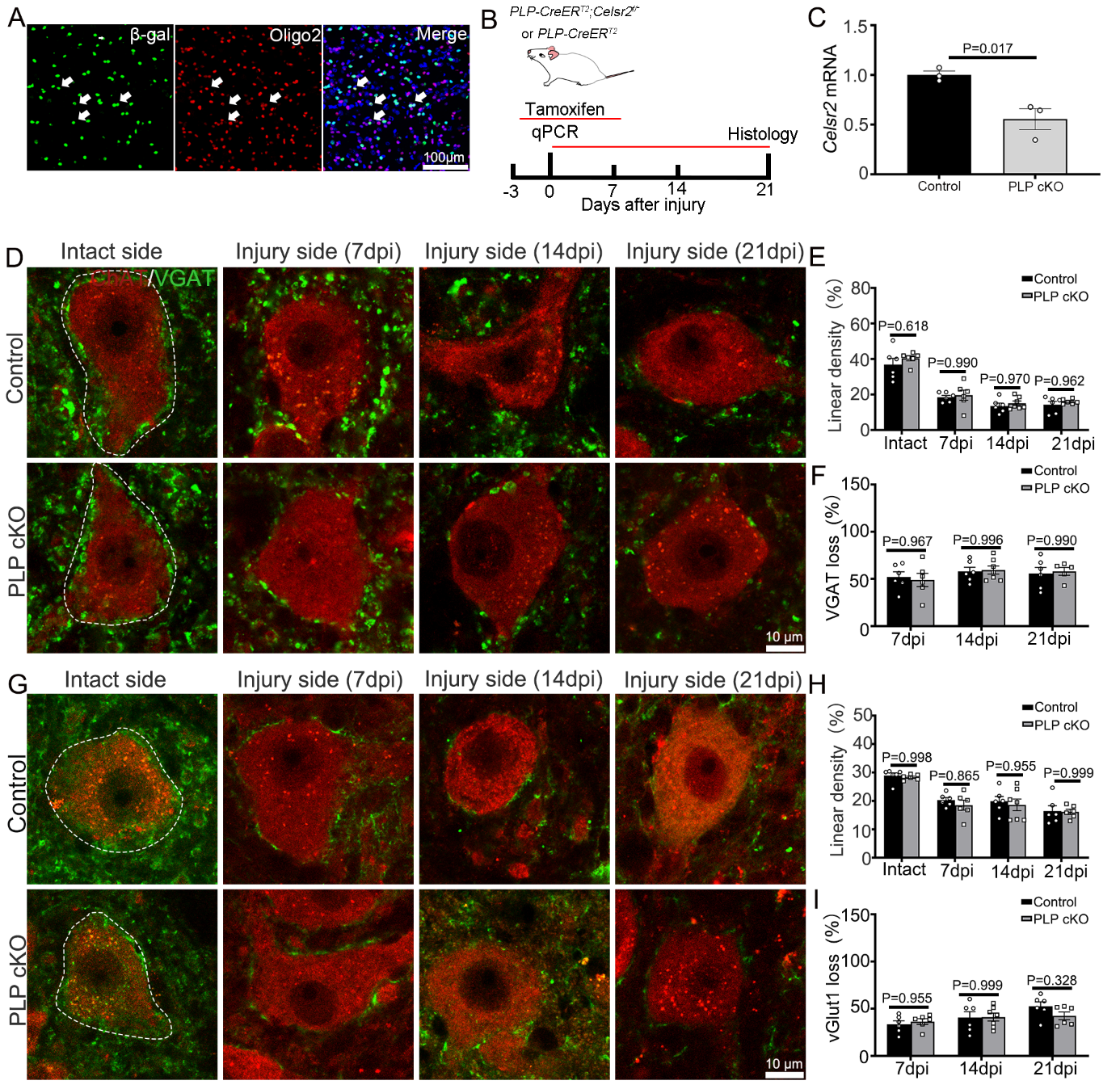
**

**Supplementary Fig. 4 Conditional inactivation of *Celsr2* in oligodendrocytes does not affect synaptic withdrawal after BPA.**

A-C: In adult *Celsr2^LacZ^* mouse, double immunostaining shows a subset of Oligo2-positive cells co-express *ß*-gal in the transverse spinal section (arrows, A). Conditional inactivation of *Celsr2* in oligodendrocytes and the experimental design are illustrated in the schematic (B). *Celsr2* mRNA levels in spinal samples are significantly downregulated in *Plp–CreER^T2^;Celsr2^f /−^* mice (*Plp* cKO) upon 3-days tamoxifen induction (C).

D-F: The coverage of inhibitory synaptic vesicles on spinal motoneuron membranes is visualized by anti-VGAT and -ChAT double immunostaining (D). The linear densities of VGAT-positive vesicles on both sides and the percentage of VGAT-positive vesicles loss (normalized to intact sides) on injury sides show similar changes in two groups (E, F; *P*>0.05).

G-I: Anti-vGlut1 and -ChAT double immunostaining shows BPA-induced excitatory synapse withdraw on spinal motoneuron membranes in two groups (G). Statistic shows the similar reduction in two groups at day 7, 14 and 21 after BPA (H, I; *P*>0.05).

Two-way ANOVA with Sidak’s multiple comparisons, n=6 mice at each timepoint in each group; dpi, days post-injury.
